# Sex-Based Differences in the Association between Nutrition Label Awareness and the Prevalence of Atopic Dermatitis: A Cross-Sectional Survey

**DOI:** 10.3390/healthcare8030210

**Published:** 2020-07-13

**Authors:** Soo Jin Kwon, Yoonjung Kim

**Affiliations:** 1 Department of Nursing, Chung-Ang University, 84 Heukseok-ro, Dongjak-gu, Seoul 06974, Korea; soojinyk@gmail.com; 2 Red Cross College of Nursing, Chung-Ang University, 84 Heukseok-ro, Dongjak-gu, Seoul 06974, Korea

**Keywords:** adult, atopic dermatitis, nutrition awareness, nutrition labels, sex

## Abstract

Atopic dermatitis is a chronic allergic disease with multifactorial causation. Although its association with diet has been demonstrated, it remains unclear whether the prevalence of atopic dermatitis among adults is associated with nutrition label awareness. Nutrition label awareness indicates knowledge of the existence of nutrition labels on processed food, and the use of them for food selection. In this cross-sectional study, we analyzed the relationship between nutrition label awareness and the prevalence of atopic dermatitis among men and women using data from the Korean National Health and Nutrition Examination Survey VI (2013–2015), including a nationally representative sample of 13,505 Korean adults (aged > 19 years). The relationship between the prevalence of atopic dermatitis and nutrition label awareness was evaluated using *t*-tests, χ^2^ tests and multivariate adjusted logistic regression analysis. Although univariate analysis showed that atopic dermatitis was associated with nutrition label awareness in both men and women, after adjustment for covariates, there was no significant association among men. The significant association between the prevalence of atopic dermatitis and nutrition label awareness among women reveals a sex-based difference in this relationship in adults, and atopic dermatitis may be managed and prevented among women by targeted education regarding nutrition labels and diet.

## 1. Introduction

Atopic dermatitis is a chronic allergic disease that is usually characterized by itching. Although it commonly affects children, atopic dermatitis can persist into adulthood or occur at older ages [1]. The prevalence of atopic dermatitis is approximately 2–10% among adults, and a recent upward trend in allergic diseases has been observed both in Korea and internationally [2,3]. Atopic dermatitis has social, economic and psychological effects [4]. Approximately 30% of individuals with severe atopic dermatitis have life-long symptoms that can significantly affect mental health and alter social performance [5]. Atopic dermatitis is commonly associated with psychological impacts [6], including mental health problems such as depression, anxiety and suicidal ideations; these problems are caused by the potential reduction in the quality of life of atopic dermatitis patients [7]. Moreover, atopic dermatitis results in financial burdens for individuals, families, and society through direct medical costs and reduced productivity [8]. Therefore, developing methods for preventing and managing atopic dermatitis is important [9,10].

Atopic dermatitis is a multifactorial condition involving complex interactions between genetic and environmental factors [2,11,12]. Among the environmental factors, diet may be an important factor because there is evidence that it has preventive and therapeutic functions [13]. A previous study focused on eliminating potential antigens for food allergies (e.g., milk, eggs, peanuts, wheat, and beans) [14], whereas recent studies focused on investigating foods that can be used to protect individuals from, or suppress, atopic dermatitis (e.g., fermented foods, vegetables, and fruit) [15,16]. People with atopic dermatitis need to pay attention to food supplements such as cow’s milk, hen’s eggs, peanuts, wheat, soy, nuts and fish [17]. Because atopic dermatitis can be triggered by certain foods, patients should exercise caution when making food choices. 

Nutrition labels are intended to help people maintain healthy diets by allowing them to make informed food choices [16,18]. Having long been used in several countries, nutrition labels were introduced in Korea in 1994 [19]. Regulations concerning food labels are being continuously revised, with an increasing number of foods being required to have proper labeling [18]. People who refer to nutrition labels tend to have healthier eating habits than those who do not, because nutrition labels are hypothesized to facilitate dietary control [20].

The increasing prevalence of atopic dermatitis among Koreans is thought to be associated with the westernization of Korean eating habits. Studies have evaluated the association between atopic dermatitis and food consumption [13,21]. However, research on whether the prevalence of atopic dermatitis among adults is associated with nutrition label awareness has not yet been conducted. Therefore, in the present study, we aimed to evaluate this association among Korean adults using representative sample data from the Korea National Health and Nutrition Examination Survey VI (2013–2015), and to assess differences associated with sex and clinical characteristics.

## 2. Materials and Methods 

### 2.1. Design and Sample

Established in 1998, the Korea National Health and Nutrition Examination Survey is managed by the Korean Centers for Disease Control and Prevention and evaluates health and nutrition in Korea. Korea National Health and Nutrition Examination Survey studies are nationally representative cross-sectional surveys that target community-dwelling Korean people. A stratified, multistage, clustered probability design is used to target the entire country and ensure that the results are nationally representative. In this study, we used data of Korean adults who had completed the Korea National Health and Nutrition Examination Survey VI between 2013 and 2015. Of 22,948 participants, 18,034 were aged > 19 years. After excluding 4529 participants with missing values or unconfirmed questionnaire responses, the final analysis included data of 13,505 participants (5481 men and 8024 women).

### 2.2. Measures

During the survey, the participants were asked whether a physician had diagnosed them with atopic dermatitis, and they were categorized based on their responses (yes/no). Furthermore, the participants were asked whether they were aware of nutritional labels, after which they were further categorized according to their responses (yes/no). Nutrition label awareness indicates knowledge of the existence of a nutrition label on processed food, and using it during food selection [22,23].

Covariates included age, sex, education, marital status, economic status, body mass index, area of residence, employment status, smoking, alcohol consumption, physical activity, diabetes and hypertension. Education level was categorized as follows: less than elementary school, middle-school graduate, high-school graduate, or university or higher degree. Marital status was categorized as unmarried or married. Economic status was defined based on household income and categorized into quartiles according to national values (average household income/$\sqrt{\mathrm{number}\;\mathrm{of}\;\mathrm{household}\;\mathrm{members}}$). Weight and height were used to calculate the body mass index (kg/m^2^). The area of residence was classified as urban or rural. Employment status was categorized as employed or unemployed. Smoking was classified as self-reported smoker or non-smoker. Alcohol consumption was classified, based on self-reported daily ethanol consumption, as mild (1–15 g/day), moderate (15–30 g/day) or heavy (>30 g/day) [24]. Physical activity was categorized as “yes” (self-reported moderately intense physical activity for ≥20 min, ≥5 days/week) or “no” [25]. The presence or absence of diabetes and hypertension was evaluated based on self-reports.

### 2.3. Statistical Analysis

Continuous data are presented as mean ± standard error (SE), whereas categorical data are presented as percentages (SE). The SAS survey procedure (version 9.3; SAS Institute Inc., Cary, NC, USA) was used for the complex sample design, which provided Korea National Health and Nutrition Examination Survey sampling weights and nationally representative estimates. T-tests and χ^2^ tests were used to evaluate the univariate associations between participants’ clinical characteristics and nutrition label awareness. Finally, the association between nutrition label awareness and the prevalence of atopic dermatitis was analyzed using a multivariate logistic regression after adjusting for demographic variables. The reference groups for comparisons included Korean men or women with nutrition label awareness. Differences were considered statistically significant at *p*-values < 0.05.

### 2.4. Statement of Ethics

This study was conducted according to the guidelines of the Declaration of Helsinki, and all procedures involving human subjects were approved by the Research Ethics Committee of the Korean Centers for Disease Control and Prevention (2013-07CON-03-4C, 2013-12EXP-03-5C). Written informed consent was obtained from all study participants. 

## 3. Results

### 3.1. Characteristics of Adults with Atopic Dermatitis According to Sex

The participants’ characteristics according to sex and atopic dermatitis status are shown in Table 1. Atopic dermatitis was observed in 98 men (1.8%) and 124 women (1.5%). Among men, atopic dermatitis was significantly associated with young age, single status, unemployment and hypertension. Among women, atopic dermatitis was significantly associated with young age, single status, smoking and alcohol consumption.

### 3.2. Associations between Atopic Dermatitis and Nutrition Label Awareness According to Sex

Associations between the prevalence of atopic dermatitis and nutrition label awareness according to sex are shown in Table 2 and Table 3. Univariate analysis revealed that atopic dermatitis was significantly associated with nutrition label awareness in both men and women.

Table 3 presents the results for the various models of associations between the prevalence of atopic dermatitis and nutrition label awareness according to sex. After adjustments for the covariates, no significant association was observed between atopic dermatitis and nutrition label awareness among men. However, among women, the prevalence of atopic dermatitis was associated with a lack of nutrition label awareness in Model 1 [odds ratio (OR): 2.24, 95% confidence interval (CI): 1.33–3.77], Model 2 (OR: 1.99, 95% CI: 1.15–3.46), and Model 3 (OR: 1.89, 95% CI: 1.08–3.3).

## 4. Discussion

The present study evaluated the association between atopic dermatitis and nutrition label awareness according to sex. Since the 1980s, the consumption of processed foods has increased, and the dietary patterns in Korea have changed. These changes are strongly correlated with an increase in the prevalence of atopic dermatitis [16]. Nutrition labels can help inform individuals of changes in the nutritional value of foods. Appropriate use of nutrition labels will eventually have a positive effect on atopic dermatitis. Individuals with chronic illnesses are more aware of nutrition labels than healthy individuals; in this population, nutrient intake is associated with nutrition label awareness [26,27]. These findings highlight the importance of nutrition education and management, as factors that can aid in disease management. As the present study only evaluated nutrition label awareness based on yes/no responses, further research is needed to provide more detailed information on the association between nutrition labels and atopic dermatitis.

Our results confirmed the previous findings that atopic dermatitis generally affects young individuals [28]. Additionally, recent international cross-sectional studies in the United States, Canada, France, Germany, Italy, Spain, the United Kingdom and Japan have shown a low prevalence of atopic dermatitis in older age groups [29]. Furthermore, our findings are concordant with those of a previous report that concluded that atopic dermatitis is associated with being single [30]. Few studies have assessed the relationship between marital status and atopic dermatitis; therefore, direct explanation is difficult. However, the average age of a single person is younger than that of a married person, suggesting that the incidence of atopic dermatitis decreases with increasing age [30]. The 2008 data from the Korea National Health and Nutrition Examination Survey showed that education, economic status and urban residence are associated with atopic dermatitis [2]. In a 2008–2013 data analysis, economic status and residential area were not related to atopic dermatitis [31]. In this study, education, economic status and urban residence were irrelevant. The present study showed that unemployment was significantly associated with atopic dermatitis among Korean men, which is consistent with previous studies reporting an association between occupation and dermatitis [32]. As time passes, the environment and circumstances of the society in Korea may change; therefore, further analysis of factors affecting atopic dermatitis is needed. In addition, further studies should evaluate this association, as sex-based differences in atopic dermatitis rates may exist because of cultural and environmental factors. 

A recent study showed that atopic dermatitis is more common among adults who drink alcohol, smoke, are obese, or have hypertension or diabetes, than among those who do not fall into these categories [6]. Alcohol consumption is associated with increased serum concentrations of immunoglobulin E, which is known to cause atopic dermatitis [6] and may explain this association. One study suggested that smoking is not associated with atopic dermatitis among Korean adults [30]. However, it is difficult to compare these observations with our results because the study did not involve a sex-specific analysis. Interestingly, one study revealed a significant association between metabolic syndrome and atopic dermatitis in women, whereas no significant association was observed in men [33]. Similarly, we found that diabetes was associated with atopic dermatitis among women only. However, as a sex-specific analysis was not performed in most of these studies, additional studies are needed in order to evaluate the sex-specific prevalence of atopic dermatitis and its association with demographic and clinical factors. 

In the final adjusted model of our study, atopic dermatitis was not found to be significantly associated with nutrition label awareness among Korean men. However, it was found to be significantly and independently associated with lack of awareness about nutrition labels among Korean women. Consumption of certain foods is associated with atopic dermatitis; the condition can be prevented by avoiding foods that cause allergic reactions, or it can be improved by eating foods that can alleviate symptoms [6]. Atopic dermatitis patients reported improved skin when excluding white flour products, gluten and nightshades from their diet and adding vegetables, organic foods and fish oil [34]. It is possible that many people are aware of the existence of nutrition labels, but such awareness does not translate into knowledge regarding “good” or “bad” food ingredients/substances. Recognizing “good” or “bad” food ingredients/substances requires additional knowledge about proteins, fats, sugars and vitamins, and their daily requirements, which depend on factors such as sex, age and physical activity. We think that specific education and guidance will be needed to raise awareness regarding nutrition labels in people, and to make their use practical as well.

The absence of a significant association between atopic dermatitis and nutrition label awareness among Korean men may be a result of traditional Korean gender roles, wherein Korean women typically handle grocery shopping. Furthermore, previous research confirmed that women are more likely to read nutrition labels than are men [35]. In addition, research has confirmed that men have a lower frequency of nutrition label use, less awareness, and less knowledge than women do [16,29]. Education regarding nutrition label awareness should consider these sex-based differences. In particular, a study is needed to raise awareness and verify the effectiveness of education on the use of nutrition labels among Korean men. Since 2018, labeling and labeling patterns have made it easier for consumers to know and understand the nutritional content of food [36]. Therefore, it is necessary to conduct research on nutrition label changes post-2018.

The major strengths of the present study are the large representative sample and sex-specific analysis. However, this study had some limitations. First, a self-reported health questionnaire was used; thus, there may be some bias as a function of the participant’s personal characteristics or circumstances, as well as recall bias, because participants respond to these surveys based on their memories. Second, the temporal relationship between the prevalence of atopic dermatitis and nutrition label awareness is not provided. Third, the cross-sectional study design precludes any commentary regarding causality in the association between atopic dermatitis and nutrition label awareness. Further longitudinal studies are needed to address these issues. Nevertheless, the present study is the first to clearly show a sex-specific association between atopic dermatitis and nutrition label awareness among Korean adults.

## 5. Conclusions

The present study showed that atopic dermatitis is independently associated with nutrition label awareness among Korean women, but not among Korean men. The results of this study suggest that education and management programs regarding atopic dermatitis should specifically target Korean women. Furthermore, we hope that these findings will be useful for designing policies and programs for the prevention and management of atopic dermatitis in the Korean population.

## Figures and Tables

**Table 1:** Differences in the prevalence of atopic dermatitis according to sex and clinical characteristics.

Characteristic		Men (*n* = 5481)	*p*-Value		Women (*n* = 8024)	*p*-Value	
		Atopic Dermatitis			Atopic Dermatitis		
		No (*N* = 5383) Mean ± SE or % (SE)	Yes (*N* = 98) Mean ± SE or % (SE)		No (*N* = 7900) Mean ± SE or % (SE)	Yes (*N* = 124) Mean ± SE or % (SE)	
Age (years)		45.35 ± 0.28	34.3 ± 1.43	<0.001	47.04 ± 0.28	33.42 ± 1.41	<0.001
Education (≤ high school)		60.35 (0.97)	63.07 (5.8)	0.645	66.06 (0.82)	59.65 (5.14)	0.194
Economic status	Q1	24.63 (0.89)	24.93 (4.92)	0.928	23.83 (0.74)	29.67 (4.57)	0.308
	Q2	25.53 (0.84)	27.47 (5.04)		25.19 (0.69)	17.85 (3.77)	
	Q3	24.41 (0.74)	25.32 (5.1)		25.12 (0.67)	27.52 (4.88)	
	Q4	25.42 (0.95)	22.28 (4.6)		25.86 (0.9)	24.96 (4.45)	
Married (yes)		73.08 (0.87)	40.59 (5.19)	<0.001	83.05 (0.67)	44.31 (4.84)	<0.001
Employed (yes)		74.9 (0.78)	64.26 (5.47)	0.033	50.42 (0.72)	54.87 (5.08)	0.388
Urban residence		82.44 (1.58)	84.49 (4.11)	0.619	84.65 (1.37)	89.86 (2.69)	0.093
Smoker (yes)		39.75 (0.84)	37.63 (5.46)	0.703	5.22 (0.32)	14.71 (3.77)	<0.001
Alcohol consumption (g/day)	0 g	26.36 (0.73)	26.92 (5.06)	0.592	57.55 (0.72)	44.03 (4.87)	0.008
	0–15 g	52.48 (0.87)	58.11 (5.96)		39.7 (0.69)	48.16 (4.94)	
	15–30 g	14.65 (0.6)	10.27 (3.43)		1.99 (0.2)	6.27 (2.65)	
	≥30 g	6.52 (0.39)	4.7 (2.25)		0.77 (0.12)	1.54 (1.53)	
BMI (kg/m^2^)		24.34 ± 0.06	24.94 ± 0.42	0.161	23.2 ± 0.05	22.8 ± 0.39	0.300
Physical activity (yes)		12.69 (0.58)	9.5 (3.61)	0.441	9.47 (0.44)	5.88 (2.08)	0.172
Hypertension (yes)		16.98 (0.57)	7.79 (2.45)	0.008	17.61 (0.54)	8.28 (2.25)	0.003
Diabetes (yes)		7.52 (0.36)	6.88 (2.6)	0.813	6.39 (0.31)	1.77 (1)	0.013

BMI, body mass index; Q1–Q4, quartiles 1–4; SE, standard error.

**Table 2:** Differences in the prevalence of atopic dermatitis according to nutrition label awareness and sex.

	Nutrition Label Awareness	Atopic Dermatitis		*p*-Value
		No % (SE)	Yes % (SE)	
Male sex	Yes	74.2% (0.71)	89.12% (3.53)	0.003
	No	25.8% (0.71)	10.88% (3.53)	
Female sex	Yes	80.59% (0.62)	87.66% (2.87)	0.040
	No	19.41% (0.62)	12.34% (2.87)	

SE, standard error.

**Table 3:** Association between nutrition label awareness and atopic dermatitis according to sex.

	Nutrition Label Awareness	Atopic Dermatitis, OR (95% CI)			
		Not Adjusted	Model 1	Model 2	Model 3
Male sex	Yes	1	1	1	1
	No	0.35 (0.17–0.72)	0.73 (0.34–1.55)	0.66 (0.29–1.46)	0.66 (0.29–1.46)
Female sex	Yes	1	1	1	1
	No	0.58 (0.35–0.98)	2.24 (1.33–3.77)	1.99 (1.15–3.46)	1.89 (1.08–3.3)

CI, confidence interval; OR, odds ratio; Male sex: Model 1 adjusted for age; Model 2 adjusted for age, marital status, and employment; and Model 3 adjusted for age, marital status, employment and hypertension; Female sex: Model 1 adjusted for age; Model 2 adjusted for age and marital status; and Model 3 adjusted for age, marital status, smoking, drinking, hypertension and diabetes.
